# Psychometric properties of the questionnaire of cognitive and affective empathy in a Portuguese sample

**DOI:** 10.1371/journal.pone.0197755

**Published:** 2018-06-01

**Authors:** Andreia Queirós, Eugénia Fernandes, Renate Reniers, Adriana Sampaio, Joana Coutinho, Ana Seara-Cardoso

**Affiliations:** 1 Neuropsychophysiology Lab, CIPsi, University of Minho, Braga, Portugal; 2 Institute of Clinical Sciences, University of Birmingham, Birmingham, United Kingdom; 3 Institute for Mental Health, University of Birmingham, Birmingham, United Kingdom; 4 Division of Psychology and Language Sciences, University College London, London, United Kingdom; Universitat Wien, AUSTRIA

## Abstract

Empathy is an important concept in psychology and cognitive neuroscience. Despite the controversy around its definition, most researchers would agree that empathy is a multidimensional phenomenon which involves a vicarious experience of another person’s affective state and an understanding of another person’s affective experience. Self-report measures of empathy constitute an important tool for both research and clinical practice. The main goal of this study was to adapt and study the psychometric properties of the Questionnaire of Cognitive and Affective Empathy (QCAE), a worldwide used measure of empathy, in a Portuguese community sample (N = 562). Confirmatory factor analyses supported the factor structure of the original QCAE. Results show that the Portuguese version of the QCAE has sound psychometric properties, with good structural validity and internal consistency for both scales (i.e., affective and cognitive) and respective subscales of the instrument (i.e., Emotion Contagion, Proximal Responsivity, Peripheral Responsivity, Perspective Taking and Online Simulation). We tested both a five correlated factor structure (Model 1) and a second-order model that postulates the affective and cognitive dimensions (Model 2). Our results show that while both models present acceptable goodness of fit indices, Model 1 performs slightly better. In conclusion, the Portuguese version of the QCAE may prove a useful tool for future cross-cultural assessments of empathy in both research and clinical practice.

## Introduction

Empathy is an ubiquitous concept that has met with increasing interest from several basic and applied fields. It has steadily permeated the speeches of politicians (e.g., Barack Obama’s 2006 speech), business leaders and other public figures (e.g., Meryl Streep’s 2017 Golden Globes speech). It is a term now firmly embedded in the lexicon of business, finance and marketing fields. Its positive social impact is heralded in books targeting the general public, and thousands of training programs have been outlined in order to instill empathic behavior in health practitioners and other social service professionals [1].

Unsurprisingly, the concept of empathy also draws immense research interest. Research has shown that empathy is strongly associated with general wellbeing and social functioning [2, 3] and is a critical factor for appropriate prosocial behavior [4]. Furthermore, empathy seems to be altered in a number of serious psychopathologies, including psychopathy [5–10], autism [11], social phobia [12], schizophrenia [13], depression [14] and borderline personality [15].

Just as other social science constructs, the conceptualization of empathy has evolved alongside the historically predominant perspectives in psychology and social sciences (for a review, see [1]). Even nowadays, conceptually distinct phenomena may be clustered under the same broad term that is empathy [16]. This lack of consensus as to its precise definition and as to its constitutive components poses a challenge to the study of empathy [17]. Nonetheless, most researchers would agree that empathy is a multidimensional phenomenon, which involves a vicarious experience of another person’s affective state and an understanding of another person’s affective experience (e.g.[18–22].

The emergence and advances of cognitive neuroscience have helped to shed light on the neurophysiological underpinnings of empathy in humans [23], pinpointing a complex network of neural regions and autonomic processes involved in the experience of empathy [10, 19, 24, 25]. Neuroscience findings indicate that empathy comprises a number of dissociable, but interacting, cognitive components, subserved by distinct, but interacting, neural networks [5, 26–28]. Many authors have thus adopted a two-component model of empathy, defining empathy as a multidimensional structure [18, 19, 29] that encompasses the ability to vicariously experience their emotional experience (affective empathy) and to comprehend other peoples’ experience (cognitive empathy) [29].

In the study of empathy, reliable self-report instruments are essential due to their relative good cost-effectiveness. By resorting to a questionnaire, a single researcher may be able to assess larger samples in a fast and collective manner. Furthermore, with this method, repeated measurements (e.g., longitudinal studies) are more easily implemented. The four most frequently used questionnaires of empathy in research are Hogan’s Empathy Scale (HES; [30]), Mehrabian and Epstein’s Emotional Empathy Scale (EES; [31], Davis’ Interpersonal Reactivity Index (IRI; [32]), and Baron Cohen’s Empathy Quotient (EQ; [33]]. However, these self-report instruments present some shortcomings. For example, most do not allow the distinction between affective and cognitive components of empathy, or their broader definitions hinder the disentanglement of empathy from related but distinct constructs, such as empathic concern or sympathy [29].

Recently, in an attempt to overcome some of these shortcomings, Reniers and colleagues [29] created the Questionnaire of Cognitive and Affective Empathy (QCAE), a novel self-report measure of empathy that takes into account the multidimensional nature of the construct as described above. The QCAE has been used worldwide [e.g. [34–37], showing good reliability and factor structure. The QCAE seems to be a psychometrically sound measure of empathy, both in the general population and in the context of clinical conditions (e.g., schizophrenia [38–41]; and psychopathy [42, 43]).

With the present study we aimed to analyze the psychometric properties of the QCAE in a large Portuguese community sample. This is important for a number of reasons: 1) to further probe the factor structure and reliability of the QCAE; 2) to further probe its validity in non-English-speaking cultures; and 3) to make a sound self-report instrument of empathy available for research with Portuguese samples, allowing for future cross-cultural assessments.

## Materials and methods

### Participants

Participants were invited to take part in this study via email through the Communication and Image departments of Portuguese universities across the country and through social media platforms such as Facebook and LinkedIn. After providing informed consent to participate, a total of 562 Portuguese adults (413 females and 149 males) filled in a standard demographics’ questionnaire and an online version of the QCAE. Age varied between 18 and 60 years old (*M =* 27.5, *SD =* 10.32); females had a mean age of 26.28 (*SD* = 9.67) whereas males had a mean age of 31.74 (*SD =* 12.02). Males were significantly older than females, as confirmed with a Welch’s *t*-test, *t*(216) = 4.11, *p*<0.001.

### Ethics

The current study was conducted according to the principles expressed in the Declaration of Helsinki and was approved by the Ethics Committee of the University of Minho. After reading a consent statement with information about the study (e.g., voluntary participation, confidentiality/anonymity, right to withdraw) and about the research team, participants who agreed with these terms proceeded with the present study. First, they provided information about demographic variables, including sex, age, country of origin, and highest level of education achieved. Next, they were presented with the online version of the QCAE.

### Questionnaire of cognitive and affective empathy (QCAE)

The QCAE [29] is a self-report measure of adults’ cognitive and affective empathy, comprised of 31 items answered on a 4-point Likert scale ranging from *strongly disagree* (*1*) to *strongly agree* (*4*). All of the QCAE’s items were originally drawn from a variety of well-known measurements, namely the Hogan Empathy Scale (HES;[30], the Empathy Subscale of the Impulsiveness-Venturesomeness Empathy Inventory (IVE; [44], the Interpersonal Reactivity Index (IRI; [18]and the Empathy Quotient (EQ; [33].

The affective empathy dimension assesses the ability to be sensitive to and to vicariously experience another’s emotional state, whereas the cognitive empathy dimension assesses the ability to form an understanding of another’s internal emotional state. The affective empathy dimension is subdivided into *Emotion Contagion* (4 items), *Proximal Responsivity* (4 items), and *Peripheral Responsivity* (4 items). Emotion contagion is characterized by the ability to automatically mirror other’s emotional states (e.g., “I am happy when I am with a cheerful group and sad when the others are glum”); while proximal responsivity is defined as the emotional state that is elicited through the perception of a close one’s feelings and moods (e.g., “Friends talk to me about their problems as they say that I am very understanding”); and lastly, peripheral responsivity is defined by the emotional response that emerges in response to social contexts that are more socially detached to the subject (e.g., “I often get deeply involved with the feelings of a character in a film, play, or novel”).

The cognitive empathy dimension is subdivided into *Perspective Taking* (10 items), and *Online Simulation* (9 items). *Perspective Taking* consists in the ability to infer things from other’s perspective (e.g., “I am quick to spot when someone in a group is feeling awkward or uncomfortable”), whilst the latter is the attempt to imagine oneself in another’s situation and infer their emotional state (e.g., “I find it easy to put myself in somebody else’s shoes”).

Scores of each subscale are obtained by summing up the corresponding individual item scores. Summing up the scores of emotion contagion, proximal responsivity, and peripheral responsivity provides a score for the affective empathy dimension; summing up the scores of perspective taking and online simulation subscales provides a score for the cognitive empathy dimension. Lastly, summing up the scores of cognitive empathy and affective empathy provides a total score for Empathy. Scores for the five subscales of the QCAE achieved acceptable to very good internal consistency indicators in the original version (Cronbach’s alphas, α, ranging from .65 to .85; [29]).

**Fig 1:**
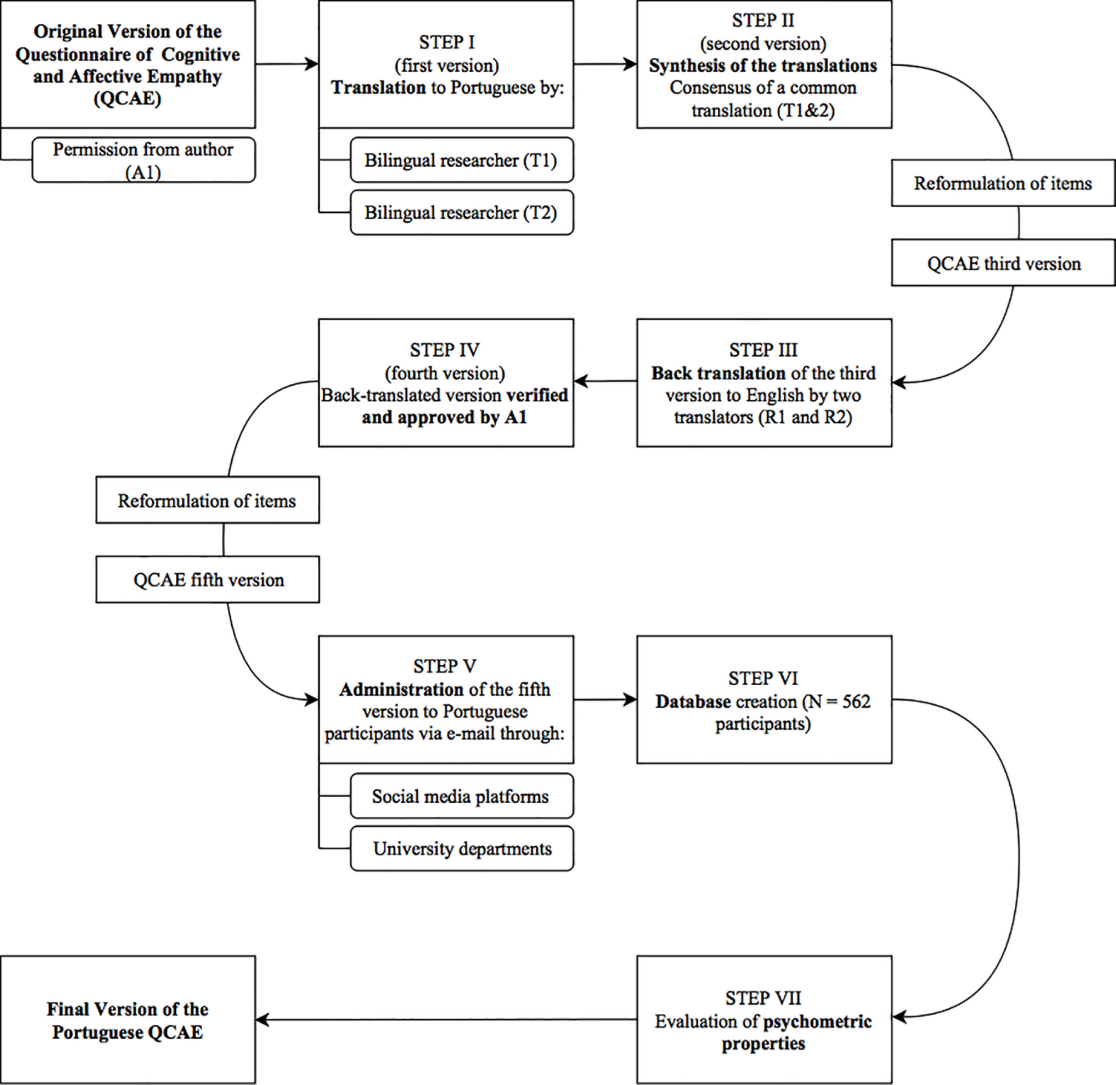
Flowchart of the Portuguese adaptation of the QCAE.

For the current study, a Portuguese version of the QCAE was created. A translation and back-translation approach was followed (Fig 1). Two English-Portuguese bilingual researchers independently translated the QCAE items from English to Portuguese and discussed their discrepancies together with a third researcher until a consensus was reached. This version was then independently back-translated to English by two other researchers (fluent in both English and Portuguese) who were unrelated to this project. The back-translated version was verified and approved by the first author of the original instrument, resulting in the Portuguese version of the QCAE, whose psychometric properties will be the focus of the current study.

### Data analysis

**Fig 2:**
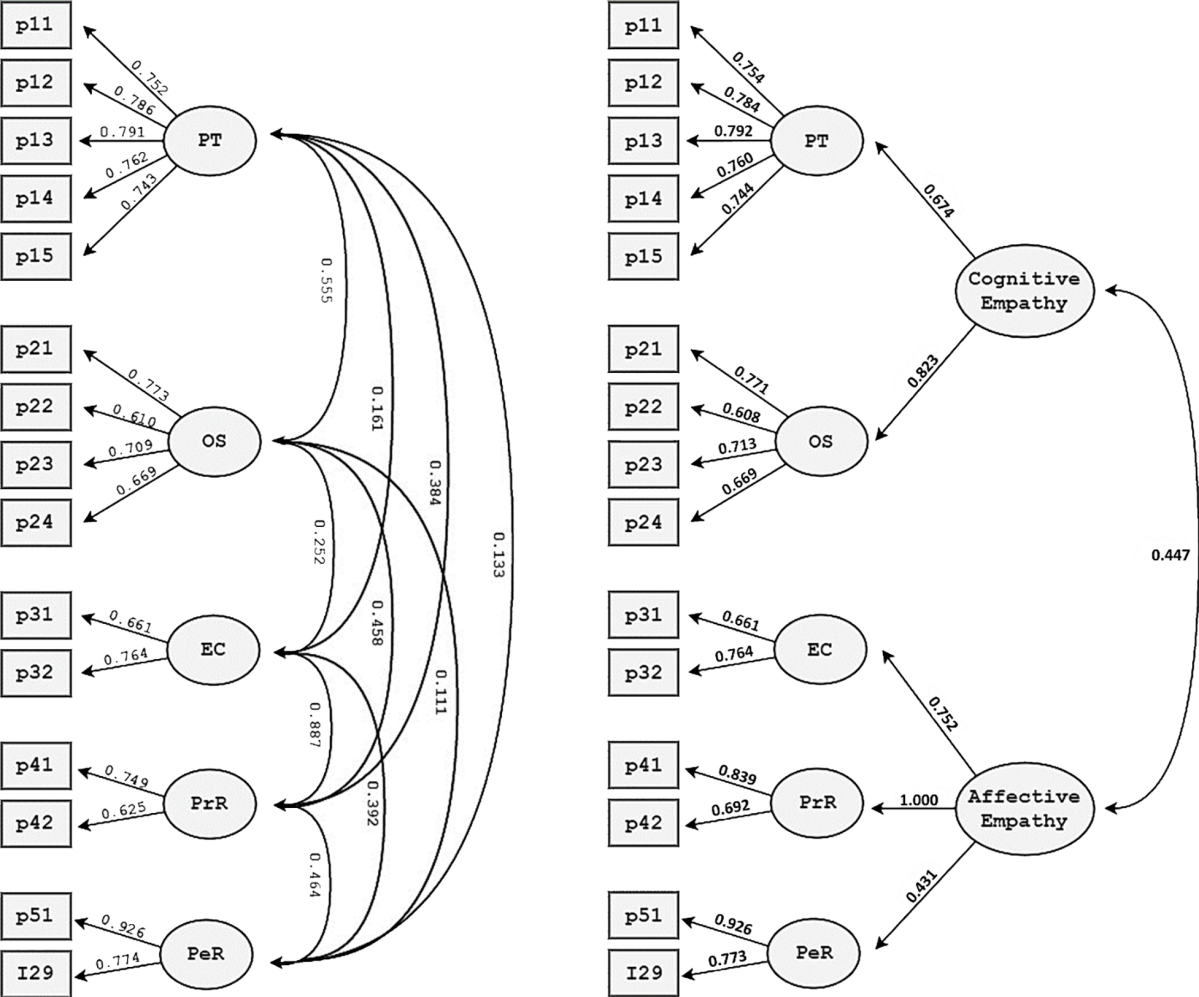
**Model 1 (left) and Model 2 (right) representing the first-order (Model 1) and the second-order (Model 2) structure of the QCEA-Portuguese version.** Boxes represent observed variables (parcels) and circles represent latent factors. Straight arrows represent factor loadings. The curved, double-headed arrows represent covariations. P21 = Parcel 1 of Factor 2 (OS), etc.; PT = Perspective taking; OS = Online simulation; EC = Emotion contagion; PrR = Proximal responsivity; PeR = Peripheral responsivity.

The present study was intended as a psychometric analysis based on the internal structure and measurement invariance of the five-factor model of the instrument for both the first- and second-order structure originally proposed by Reniers and colleagues [29] (Fig 2). Specifically, the first-order structure (Model 1) tested the parcel loadings on the five subscales of the QCAE (i.e. Emotion Contagion, Proximal Responsivity, Peripheral Responsivity, Perspective Taking, and Online Simulation). The second-order structure (Model 2) builds on Model 1 by adding the hypothesized higher-order cognitive and affective empathy constructs. Both models were assessed via confirmatory factor analysis (CFA), using R Studio version 3.2.4, through “Lavaan” package [45].

For completeness, and to obtain item loadings, we first conducted an item-level CFA analysis. Next, and similarly to the original version of the QCAE, we implemented a parceling approach to reduce the likelihood of bias in structural parameters [46, 47]. While the use of parcels to investigate factor structures might be arguable, we considered that, in the particular case of the QCAE, this was the most appropriate approach. The QCAE presents an uneven number of items per factor (e.g. the Perspective Taking subscale comprises 10 items whereas each of the Affective Empathy subscales comprise only 4 items each), making the factors differentially susceptible to different degrees of measurement error. By using parcels, because fewer indicators per construct are used, the amount of measurement error is mitigated [48]. Additionally, while the items taping a construct would probably present non normal distributions, the resulting distribution of their parcels would more likely approach the “true” distribution of the construct [48]. Finally, the main goal of the current study was to replicate, as close as possible, Reniers and colleagues’ [29] methodology and to investigate whether their proposed factor structures for the QCAE held in a Portuguese large sample.

We have, however, changed the parceling strategy used in order to reduce the likelihood of bias in structural parameters [46, 47]. Instead of averaging the highest loading pairs of items, parcels within each factor were created by averaging the items with the highest and lowest item-scale correlations. The only exception to this strategy happened with factor 5 (i.e., Peripheral Responsivity) which only had three items since item 17 was excluded from this version due to an extremely low loading value (cf. Results and Discussion for further details). In this exception, to preserve the model’s structure and the described parceling strategy, the item with intermediate item-scale correlation was selected as a second “parcel” (I29). Therefore, in the CFA analyses, only this parcel was treated as an ordered variable (refer to Table A in S1 File for a complete description of the parcels). Nonetheless, to allow for a direct comparison with the analyses conducted on the original instrument, we ran a further set of CFA analyses using Reniers and colleagues’ parceling strategy (refer to Table B in S1 File for a complete description of the parcels).

Model fit was assessed by chi-square (χ^2^) goodness of fit test, Root Mean Squared Error of Approximation (RMSEA) and the Comparative Fit Index (CFI). Although the Chi-square test is usually reported in CFA, its use has been criticized due to its sensitivity to sample size effects, which may lead to too many type I errors when variables have non-normal distributions, with a high degree of kurtosis [49]. This and other additional measures are nonetheless presented to allow for a comparison with original versions of the instrument [29]. Model fit was evaluated according to the dual-criteria method proposed by Hu and Bentler [50] which set the acceptable threshold values for CFI at ≥.90 and for RMSEA at ≤.08.

In order to assess the extent of the models’ internal consistency, Cronbach’s coefficient alpha and Composite Reliability indexes were inspected. Cronbach’s alpha is the estimator most frequently reported in the literature. However, its use has been criticized as it provides a lower bound on true reliability [51], particularly when a model is not unidimensional [52]. Thus, as an alternative, reliability was also assessed through the Composite Reliability (CR) index [53, 54]. Bagozzi and Yi [55], as well as Hair and colleagues [56] recommend CR values ≧ 0.6.

Finally, a sex invariance analysis (configural, metric, and scalar invariance) was conducted to ascertain whether the instrument validly assesses the same constructs in both genders. Lastly, sex differences in empathy scores were inspected with Welch *t*-tests which are considered to be more robust than Student’s *t*-tests [57], especially when sample sizes are unequal and thus unequal variances more difficult to be detected.

## Results

### Confirmatory factor analysis (CFA) and internal consistency

Analysis of responses indicated that the data did not follow a normal distribution (Mardia multivariate kurtosis = 39.60, *p* < .001; [58]and thus the *diagonally weighted least squares* estimation method with robust standard errors (Satorra-Bentler scaled statistic) was selected in the CFA. Item-level CFA were performed on the five-factor structure models originally proposed by Reniers and colleagues [29]. Goodness of fit measures for the two models were as follows: for Model 1, χ^2^(424) = 3466.083 *p* < .001, CFI = .910, TLI = .901, SRMR = .091 and RMSEA = .096; for Model 2, χ^2^(428) = 3413.284 *p* < .001, CFI = .908, TLI = .900, SRMR = .094 and RMSEA = .096). All item loading values were significant, with the exception of item 17, which revealed a value lower than 0.50, namely 0.023 (*p* = 0.419). A careful examination of item 17 highlighted its statistical and theoretical failure (cf. Discussion), and the item was therefore excluded from the Portuguese version of the QCAE. Following its removal, all 30 factor loadings were above .5 with *p*-values < .001 (Table A in S1 File).

Next, we followed a parceling approach to reduce the likelihood of bias in structural parameters (cf. Data Analysis). Items were parceled and the structure of the two models was assessed with a further set of five-factor CFA (Fig 1; Table C in S1 File). Of notice, and similarly to Reniers and colleagues [29], a negative residual variance was observed in the peripheral responsivity factor in Model 2; consequently, this factor was constrained to zero. All parcel loadings were above .5 and significant at *p* < .001, and total sample values ranged between .625 and .926 (Model 1) and between .608 and .926 (Model 2). In the male subsample, parcel loadings ranged between .528 and 1.054 (Model 1) and between .518 and 1.074 (Model 2). In the female subsample, parcel loadings ranged between .638 and .936 (Model 1) and between .633 and .937 (Model 2). As presented in Table 1, both Model 1 and 2 presented satisfactory values in the CFI and RMSEA goodness-of-fit indices for the total sample, as well as for separate female and male subsamples. The analyses using Reniers and colleagues’ parceling strategy provided similar results, as can be observed in Table D in S1 File.

**Table 1:** Goodness of fit tests and indices (Parcel-level analyses).

		Model 1 5 correlated factors				Model 2 5 factors with 2 correlated second-order factors			
		Original version	Portuguese version			Original version	Portuguese version		
		Total sample	Total sample	Male subsample	Female subsample	Total sample	Total sample	Male subsample	Female subsample
Goodness of fit measure	Value indicating good fit								
χ^2^	*ns*	χ^2^(80) = 193.90 *p* < .001	χ^2^(80) = 325.26 *p* < .001	χ^2^(80) = 161.25 *p* < .001	χ^2^(80) = 209.36 *p* < .001	χ^2^(85) = 244.31 *p* < .001	χ^2^(85) = 339.46 *p* < .001	χ^2^(85) = 166.49 *p* < .001	χ^2^(85) = 212.31 *p* < .001
RMSEA (90% CI)	≤.08	.067 (.055-.079)	.047 (.042-.052)	.054 (.042-.066)	.041 (.034-.048)	.077 (.066-.088)	.050 (.045-.056)	.057 (.044-.070)	.042 (.035-.050)
CFI	≥.90	.947	.979	.966	.983	.925	.975	.960	.981
TLI	≥.90	.930	.973	.956	.978	.908	.969	.951	.976
SRMR	≤.08	.030	.047	.065	.045	.042	.052	.072	.049

*Note*. χ^2^ = Chi-square goodness of fit test; RMSEA = Root mean squared error of approximation; CI = Confidence interval; CFI = Bentler’s comparative fit index; TLI = Tucker-Lewis Index; SRMR = Standardized Root Mean Square Residual. Robust χ^2^ and fit measures values are presented for the Portuguese version of the QCAE.

Regarding internal consistency, Cronbach’s alphas at the subscale and total scale levels ranged from .62 to .87, while the cognitive and affective dimensions presented alphas of .87 and .80, respectively (Table E in S1 File). Importantly, the five subscale constructs of the two QCAE models presented good composite reliability (CR) values, ranging between .643 and .913 for the complete sample (Table 2). Similarly, both dimensions and total scale presented CRs above .90. In sum, these indices, that differently quantify the same concept, provided a consistent indication of adequate reliability.

**Table 2:** Composite reliability indices obtained in the confirmatory factor analyses for the models in Fig 2.

	Model 1			Model 2		
	Total sample	Male subsample	Female subsample	Total sample	Male subsample	Female subsample
Total Score	.948	.944	.946	.950	.947	.951
Cognitive Empathy	N.A.	N.A.	N.A.	.913	.913	.912
Perspective Taking	.877	.882	.874	.877	.882	.874
Online Simulation	.786	.776	.787	.786	.776	.787
Affective Empathy	N.A.	N.A.	N.A.	.902	.881	.902
Emotion Contagion	.675	.504	.708	.675	.506	.708
Proximal Responsivity	.643	.611	.621	.742	.711	.726
Peripheral Responsivity	.842	.879	.810	.841	.887	.823

*Note*. N. A. = Not applicable

### Measurement invariance across genders

The two models were well fit both for male and female samples (Table 1), indicating configural invariance. Moreover, Model 1 presented full metric invariance across sexes (ΔCFI = -0.001, ΔRMSEA = 0.002, and non significant χ^2^ differences) and partial scalar invariance (ΔCFI = -0.001, ΔRMSEA = 0.000, and nonsignificant χ^2^ differences) was found after relaxing the intercept of parcels P41, P11, P52, and P51. Model 2 presented full metric (ΔCFI = -0.000, ΔRMSEA = -0.002, and nonsignificant χ^2^ differences) and partial scalar invariance (ΔCFI = -0.000, ΔRMSEA = -0.000, and nonsignificant χ^2^ differences) after relaxing the intercept of parcels P41, P51, P52, P31 and P11. These results suggest that both models were reliable across genders.

### Gender differences in empathy

Similar to the original study, females scored significantly higher than males on all empathy measures. Independent-samples Welch’s *t*-test confirmed these differences: on the affective empathy scale, females had a mean of 33.46 (*SE* = 0.24) and males had a mean of 29.30 (*SE* = 0.40), *t*(560) = 8.83, *p*<0.001; on the cognitive empathy scale, females had a mean score of 59.11 (*SE* = 0.36) whereas males had a mean of 56.68 (SE = 0.67), *t*(560) = 3.38, *p*<0.05. These differences were observed across all subscales (Table 3). GLM univariate analyses (Table F in S1 File) confirmed that these differences remained significant after controlling for age, with the exception of the cognitive empathy scale (*F*(1, 558) = 2.49, *p =* .115) and its perspective taking subscale (*F*(1, 558) = .334, *p =* .563).

**Table 3:** Welch’s two sample *t*-test results comparing males and females on QCAE scores.

	Males N = 149		Females N = 413				
	M	SD	M	SD	Welch’s *t*-test	Cohen’s *d*	Hedges’ *g*
Total QCAE score	85.98	10.74	92.57	9.65	*t*(563.04) = -204.77, *p<*0.001	0.65	0.66
Cognitive Empathy	56.68	8.21	59.11	7.27	t(564.79) = -178.04, *p<*0.001	0.31	0.32
Perspective Taking	29.44	5.49	30.98	4.64	*t*(570.02) = -140.47, *p<*0.001	0.30	0.32
Online Simulation	27.24	4.17	28.13	3.91	*t*(574.72) = -157.07, *p<*0.001	0.22	0.22
Affective Empathy	29.30	4.94	33.46	4.92	*t*(568.94) = -139.84, *p<*0.001	0.84	0.84
Emotion Contagion	10.81	2.22	12.09	2.22	*t*(602.81) = -106.69, *p<*0.001	0.58	0.58
Proximal Responsivity	11.09	2.24	12.44	2.02	t(607.91) = -116.38, *p<*0.001	0.63	0.65
Peripheral Responsivity	7.41	2.38	8.93	2.13	*t*(602.47) = -73.617, *p<*0.001	0.67	0.69

Note. N = sample size, M = Mean, SD = Standard Deviation.

## Discussion

In the current study, the psychometric properties and validity of a Portuguese version of the QCAE, a recently developed self-report measure of empathy, were tested in a large community sample. Our goals were two-fold. We wanted to further probe the factor structure and reliability of the QCAE and probe its validity in non-English-speaking cultures. Most importantly, we wanted to make a sound self-report instrument of empathy available for research with Portuguese samples, allowing for future cross-cultural assessments. Here, we evaluated the internal structure of the five-factor models (a first-order structure and second-order structure) originally proposed by Reniers and colleagues [29] with a confirmatory factor analysis (CFA) procedure. Our results indicate that the Portuguese QCAE has sound psychometric properties, good structural validity with acceptable goodness-of-fit indices and internal consistency across the scales and subscales of the instrument.

Currently most researchers would agree that empathy is a multidimensional phenomenon, which involves a vicarious experience of another person’s affective state (i.e. affective empathy) and an understanding of another person’s experience (i.e. cognitive empathy) [e.g. [18–22]. Yet, most available measures of empathy do not reflect this definition. For example, some do not distinguish between the affective and cognitive dimensions, and some adopt broader definitions of related but distinct constructs, such as empathic concern or sympathy [29]. This distinction is particularly important for disentangling putative neurocognitive impairments commonly associated with a number of psychopathologies. For example, individuals with high levels of psychopathy, who are known to show little concern for their victims but are highly manipulative, have been found to present deficits in affective empathy but intact cognitive empathy abilities [59, 60]. In contrast, individuals with high autistic traits, who are characterized by problems with social interaction and communication, seem to show impairments in cognitive empathy abilities but not in affective empathy. This current and multidimensional definition of the construct of empathy is patent in the original QCAE. Our results demonstrate that the Portuguese version of the QCAE presents a similar internal structure making it a reliable measurement of empathy and its distinct dimensions in Portuguese samples.

All the items of the Portuguese QCAE presented good factor loadings, except for item 17 from the peripheral responsivity subscale (“It is hard for me to see why some things upset people so much”). This item revealed an extremely low loading value and had to be excluded from the Portuguese version. The three remaining items that compose this subscale (items 2, 11 and 29) address emotional responses to social contexts that are detached to the subject, such as narrative works of art (movies, plays, novels). Item 17, on the other hand, pertains to an even more detached and non-specific social context. Taken together, this evidence suggests a theoretical failure of item 17 in the Portuguese QCAE and this item was thus removed from the final version of the instrument. In fact, the peripheral responsivity subscale has been pointed out as one of the fragilities of the QCAE by authors who recently applied the measure to clinical samples [39, 41] and found that both the reliability and convergent validity of this subscale was not very satisfactory. As argued by these authors, the fact that the items that compose the peripheral responsivity scale focus on our response to the feelings of others in a detached social context, such as characters in movies, may be confusing or not very relevant to some individuals. Moreover, three out of four items included in this scale are reverse-coded and the wording of these items may be more confusing to the reader.

Another criticism that has been pointed out to the QCAE has to do with the label attributed to one of its cognitive empathy subscales called “online simulation”. This label may be misleading as an index of cognitive empathy because in social neuroscience simulation is traditionally used to refer to the automatic mirroring processes associated with affective empathy [e.g.[61].

The present study is not without its limitations. One of them is the unequal distribution of male and female participants, which could have biased our results. Our finding of measurement invariance across genders suggests that the same constructs are being measured across the two groups and, because partial scalar invariance was achieved, comparisons of the latent means across groups can be conducted [62]. However, due to unequal sized groups of male and female participants, it is possible that the extent of non-invariance was underestimated. We consider, though, that this shortcoming is to some extent overcome by our overall results. First, both first- and second-order structures originally proposed by Reniers and colleagues [29] present acceptable goodness-of-fit indices in our complete sample and in gender-specific samples, thus indicating that data from both samples are well described by these models. Second, consistent with previous self-report studies on empathy (e.g.,[63–65], between gender-comparison analyses revealed that females scored significantly higher than males on most empathy subscales, even after controlling for age.

Another limitation is the absence of convergent validity tests of the QCAE with other measures of empathy. This was due to a lack of a validated Portuguese instrument assessing the same multidimensional conceptualization of empathy. A further concern, which is transversal to all self-report studies on empathy, is that self-report scores might not reflect actual abilities nor predict actual empathic responses in everyday situations. Future studies could include behavioral tasks of affective empathy, such as the *Empathy for emotional facial expressions task* [10, 42, 60]. This task presents strong correlations with other measures of empathy and related constructs, and is sensitive to individual differences in psychopathic traits in the general population [42, 59, 60]. Importantly, behavioral performance in this task has been shown to be modulated by brain regions deemed to be crucial for the generation of affective empathic responses [10]. Similarly, future studies could test for convergent validity of cognitive empathy by including measures of empathic accuracy that evaluate empathy as a performance variable rather than a self-report variable [66]. This is important because, as pointed out before, one’s evaluation of one’s ability to infer other people’s thoughts and feelings might be distinct from one’s actual ability [67].

Finally, although the structure of two correlated higher order factors of cognitive and affective empathy (Model 2) was still considered acceptable, the five correlated factor structure (Model 1) presented a slight advantage based on the examination of the goodness of fit indices. These results, which provide a stronger support for the five correlated factor model, are in line with recent data from Myszkowski and colleagues [68]. Additionally, similarly to both the original version of the QCAE and to the more recent French adaptation of the instrument [68], the two correlated higher order factor model presented a negative residual variance problem in the Peripheral Responsivity factor. As has been pointed out by Chen and colleagues [62], among the possible causes for improper solutions are, for example, sample size fluctuations, outliers or influential cases, or even model misspecification. Future studies should seek to more fully determine the conditions under which negative estimates of error variance occur in the QCAE, for example, by examining the contribution of overall sample size and a more proportionate distribution of gender sample sizes.

Nevertheless, the goals of this study were fulfilled. The analyses of the psychometric properties of the Portuguese version of the QCAE provide further support of the original QCAE factor structure and reliability. Our results also indicate that this is a reliable, valid, and structurally sound measure of empathy which can be used in Portuguese samples. Self-report instruments measuring empathy in the general Portuguese population are scarce and a necessity given the increasing research and clinical practice interest in this construct. Plus, they are essential for efficient research. They are inexpensive, easy to assess and easy to use. Self-report instruments allow a single researcher to assess larger samples in a fast and collective manner. Hopefully, the availability of a sound self-report instrument will enrich research on empathy in Portugal, providing an important tool for the assessment of empathy as a multidimensional construct and allowing the possibility of future multicultural assessments. A sound measure of empathy is important not only for the study of individual differences and for the disambiguation of impairments in empathy in distinct psychopathologies (e.g., psychopathy, autism spectrum disorder), but also for the screening of general social cognition abilities or for gathering evidence-based information on the change of empathic abilities of subjects undergoing therapeutic interventions.

## Supporting information

S1 File Supplementary materials. Items of the Portuguese QCAE; Table A (Parcels composition for Models 1 and 2); Table A (Parcels composition according to Reniers and colleagues [29] and Myszkowski and colleagues [68]); Table C (Standardized loadings for parcels in Model 1 and Model 2); Table D (Goodness of fit tests and indices, using Reniers and colleagues [29] and Myszkowski and colleagues [68] parcels composition)); Table E (Cronbach’s Alpha values in the second-order model of the QCAE); Table F (GLM Univariate analyses of QCAE scores by Sex (factor) with age (covariate)).
(DOCX)

S1 Dataset Database of the Portuguese QCAE sample. (XLS)
