# Supplementary material for: Psychometric properties of the questionnaire of cognitive and affective empathy in a Portuguese sample
Source: PLoS One. 2018 Jun 1;13(6):e0197755. doi: 10.1371/journal.pone.0197755 (PMC5983521; doi:10.1371/journal.pone.0197755)
Supplement: S1 File — Items of the Portuguese QCAE; Table A (Parcels composition for Models 1 and 2); Table A (Parcels composition according to Reniers and colleagues [29] and Myszkowski and colleagues [68]); Table C (Standardized loadings for parcels in Model 1 and Model 2); Table D (Goodness of fit tests and indices, using Reniers and colleagues [29] and Myszkowski and colleagues [68] parcels composition)); Table E (Cronbach’s Alpha values in the second-order model of the QCAE); Table F (GLM Univariate analyses of QCAE scores by Sex (factor) with age (covariate)). (DOCX) [file pone.0197755.s001.docx]

**Portuguese Version of the QCAE (Questionário de Empatia Afetiva e Cognitiva)**

**Queirós, Fernandes, Reniers, Sampaio, Coutinho, & Seara-Cardoso (2018)**

English Version: Reniers, Corcoran, Drake, Shryane, & Völlm (2011). doi: [10.1080/00223891.2010.528484](http://www.tandfonline.com/doi/abs/10.1080/00223891.2010.528484)

Por favor, indique até que ponto cada uma destas afirmações se aplica a si.

As possíveis respostas são “*Discordo Fortemente”*, “*Discordo Ligeiramente*”, “*Concordo Ligeiramente*” e “*Concordo Fortemente*”.

1. Às vezes tenho dificuldade em ver as coisas do ponto de vista de outra pessoa

2. Quando vejo um filme ou uma peça de teatro, normalmente sou objetivo(a) e não costumo envolver-me totalmente

3. Numa situação de desacordo, tento ver o lado de toda a gente antes de tomar uma decisão

4. Às vezes tento compreender melhor os meus amigos imaginando como são as coisas a partir da perspetiva deles

5. Normalmente, quando estou chateado(a) com alguém, tento por momentos pôr-me na pele dessa pessoa

6. Antes de criticar alguém, tento imaginar como me sentiria se estivesse no seu lugar

7. É frequente ficar emocionalmente envolvido(a) com os problemas dos meus amigos

8. Tenho tendência a ficar nervoso(a) quando os outras à minha volta parecem estar nervosos

9. As pessoas com quem estou têm uma grande influência no meu humor

10. Afeta-me muito quando um dos meus amigos parece estar chateado

11. Frequentemente fico profundamente envolvido com os sentimentos duma personagem dum filme, peça de teatro ou livro

12. Fico muito perturbado(a) quando vejo alguém a chorar

13. Fico alegre quando estou com um grupo de pessoas bem-dispostas e fico triste quando os outros estão em baixo

14. Preocupa-me quando os outros estão preocupados e nervosos

15. Consigo perceber facilmente quando alguém quer entrar numa conversa

16. Consigo perceber rapidamente quando alguém diz uma coisa mas quer dizer outra

17. É difícil para mim compreender porque é que algumas coisas perturbam tanto as pessoas

18. É fácil para mim pôr-me na pele de outra pessoa

19. Sou bom (boa) a prever como é que alguém se irá sentir

20. Sou rápido(a) a identificar quando, num grupo, alguém se está a sentir constrangido ou desconfortável

21. As outras pessoas dizem-me que sou bom (boa) a compreender como elas se estão a sentir e o que estão a pensar

22. Percebo facilmente se alguém está interessado ou entediado com o que estou a dizer

23. Os meus amigos conversam comigo sobre os seus problemas porque dizem que sou muito compreensivo(a)

24. Consigo sentir se estou a ser intrusivo(a), mesmo que a outra pessoa não mo diga

25. Consigo perceber facilmente aquilo de que a outra pessoa quer falar

26. Consigo perceber quando alguém está a esconder as suas verdadeiras emoções

27. Sou bom a prever o que é que alguém irá fazer

28. Normalmente, consigo compreender o ponto de vista de outra pessoa mesmo que não concorde com ela

29. Normalmente, mantenho-me emocionalmente desligado(a) quando estou a ver um filme

30. Tento sempre considerar os sentimentos da outra pessoa antes de fazer alguma coisa

31. Antes de fazer alguma coisa, tento ter em consideração como é que os meus amigos vão reagir

| **Table A.** Parcels composition for Models 1 and 2. | | |
| --- | --- | --- |
| Parcels per subscale | Item (factor loading) | |
| PT - Perspective Taking | |  |
| P11 | 20 (.778); 27 (.622) |  |
| P12 | 21 (.763); 24 (.637) |  |
| P13 | 26 (.733); 15 (.676) |  |
| P14 | 25 (.727); 22 (.676) |  |
| P15 | 16 (.696); 19 (.708) |  |
| OS – Online Simulation | |  |
| P21 | 18 (.702); 31 (.502) |  |
| P22 | 4 (.686); 1 (.524) |  |
| P23 | 30 (.680); 28 (.581) |  |
| P24 | 3 (.656); 5 (.622); 6 (.640) |  |
| EC – Emotion Contagion | |  |
| P31 | 13 (.745); 9 (.552) |  |
| P32 | 8 (.668); 14 (693) |  |
| PrR – Proximal Responsivity | |  |
| P41 | 23 (.722); 12 (.524) |  |
| P42 | 7 (.576); 10 (.606) |  |
| PeR – Peripheral Responsivity | |  |
| P51 | 2 (.756); 11 (.843) |  |
| I29 | 29 (.824) |  |

**Table B**. Parcels composition as in Reniers and colleagues [28] and Myszkowski and colleagues [72].

| QCAE subscale | Perspective Taking | Online Simulation | Emotion Contagion | Proximal Responsivity | Peripheral Responsivity |
| --- | --- | --- | --- | --- | --- |
| Parcels (P) and respective items (i) | P11 = i25 & i26  P12 = i19 & i24  P13 = i20 & i27  P14 = i16 & i22  P15 = i15 & i21 | P21 = i3 & i6  P22 = i5 & i30  P23 = i4 & i28  P24 = i18 & i31 | P31 = i13 & i14  P32 = i8 & i9 | P41 = i7 & i23  P42 = i10 & i12 | P51 = i2 & i29  P52 = i11 & i17 |

| **Table C.** Standardized loadings for parcels in Model 1 and Model 2. | | | | | | | | |
| --- | --- | --- | --- | --- | --- | --- | --- | --- |
|  | QCAE | | | | | | | |
|  | Model 1 | | |  | Model 2 | | | |
|  | Total | Sex | |  | Total | Sex | | |
| Parcels |  | Male | Female |  |  | Male | Female | |
| Perspective Taking |  |  |  |  |  |  | |  |
| P11 | .752 | .760 | .771 |  | .754 | .764 | | .773 |
| P12 | .786 | .723 | .790 |  | .784 | .722 | | .788 |
| P13 | .791 | .845 | .772 |  | .792 | .847 | | .773 |
| P14 | .762 | .727 | .763 |  | .760 | .717 | | .763 |
| P15 | .743 | .808 | .712 |  | .744 | .813 | | .713 |
| Online Simulation |  |  |  |  |  |  | |  |
| P21 | .773 | .816 | .752 |  | .771 | .815 | | .751 |
| P22 | .610 | .577 | .637 |  | .608 | .578 | | .633 |
| P23 | .709 | .662 | .718 |  | .713 | .662 | | .723 |
| P24 | .669 | .661 | .661 |  | .669 | .661 | | .661 |
| Emotion Contagion |  |  |  |  |  |  | |  |
| P31 | .661 | .528 | .703 |  | .661 | .518 | | .706 |
| P32 | .764 | .631 | .777 |  | .764 | .644 | | .774 |
| Proximal Responsivity |  |  |  |  |  |  | |  |
| P41 | .749 | .779 | .704 |  | .839 | .881 | | .795 |
| P42 | .625 | .538 | .638 |  | .692 | .589 | | .714 |
| Peripheral Responsivity |  |  |  |  |  |  | |  |
| P51 | .926 | 1.054 | .936 |  | .926 | 1.074 | | .937 |
| I29 | .774 | .688 | .702 |  | .773 | .675 | | .725 |

**Table D**. Goodness of fit tests and indices, using Reniers and colleagues [28] and Myszkowski and colleagues [72] parcels composition.

|  |  | Model 1  5 correlated factors | | |  | Model 2  5 factors with 2 correlated second-order factors | | |
| --- | --- | --- | --- | --- | --- | --- | --- | --- |
|  |  | Reniers et al. (2011) | Myszkowski et al. (2017) | Portuguese data |  | Reniers et al. (2011) | Myszkowski et al. (2017) | Portuguese data |
| Goodness of fit measure | Cut-off |  |  |  |  |  |  |  |
| χ^2^ | *ns* | χ^2^(80)=193.90  *p*<.001 | χ^2^(80)=277.33, *n.r.* | χ^2^(80)=328.35, *p* <.001 |  | χ^2^(85)=244.31  *p*<.001 | χ^2^(84)=305.24, *n.r.* | χ^2^(84)=334.06  *p*<.001 |
| Df |  | 80 | 80 | 80 |  | 85 | 84 | 84 |
| χ^2^/df | <5 | 2.42 | 3.46 | 4.10 |  | 2.87 | 3.63 | 3.98 |
| CFI | ≥.90 | .947 | .938 | .922 |  | .925 | .930 | .922 |
| SRMR | <.08 | .030 | .058 | .069 |  | .042 | .068 | .072 |
| RMSEA | ≤.08 | .067 | .076 | .074 |  | .077 | .079 | .073 |
| AIC | Smallest value | 273.897 | 9706.00 | 12786.40 |  | 314.309 | 9726.00 | 12784.12 |
| B-S boot | *ns* | *p* = .001 | *n.r.* | *p* < .001 |  | *p* = .001 | *n.r.* | *p* < .001 |
| TLI | ≥.90 | .930 | *n.r.* | .898 |  | .908 | *n.r.* | .902 |

*Note*. Maximum Likelihood with Wishart approach estimator. χ^2^ = Chi-square goodness of fit test; df = degrees of freedom; CFI = Bentler’s comparative fit index; SRMR = Standardized Root Mean Square Residual; RMSEA = Root mean squared error of approximation; CI = Confidence interval; AIC = Akaike’s Information Criterion; B-S boot = Bollen-Stine bootstrap test; TLI = Tucker-Lewis Index; *n.r.* = not reported.

**Table E.** Cronbach Alpha values in the second-order model of the QCAE (with parcels)

|  | Original version |  | Portuguese version | | |
| --- | --- | --- | --- | --- | --- |
|  | Total sample |  | Total sample | Males | Females |
|  |  |  |  |  |  |
| Full-scale | NR |  | .87 | .86 | .86 |
| Cognitive Empathy | NR |  | .87 | .87 | .87 |
| PT | .85 |  | .87 | .87 | .86 |
| OS | .72 |  | .79 | .77 | .79 |
| Affective Empathy | NR |  | .80 | .74 | .79 |
| EC | .83 |  | .69 | .60 | .70 |
| PrR | .65 |  | .62 | .62 | .59 |
| PeR | .70 |  | .80 | .81 | .77 |
|  |  |  |  |  |  |

*Note*: NR = Not reported on the original version of the instrument.

**Tables F.** GLM Univariate analyses of QCAE scores by Sex (factor) with age (covariate).

| **Tests of Between-Subjects Effects** | | | | | |
| --- | --- | --- | --- | --- | --- |
| Dependent Variable: QCAE_TOTAL | | | | | |
|  | | | | | |
| Source | Type III Sum of Squares | df | Mean Square | F | Sig. |
| Corrected Model | 4847.011^a^ | 3 | 1615.670 | 16.289 | .000 |
| Intercept | 446218.644 | 1 | 446218.644 | 4498.803 | .000 |
| Sex | 894.235 | 1 | 894.235 | 9.016 | .003 |
| Age | 31.081 | 1 | 31.081 | .313 | .576 |
| Sex * Age | 40.710 | 1 | 40.710 | .410 | .522 |
| Error | 55345.838 | 558 | 99.186 |  |  |
| Total | 4695751.000 | 562 |  |  |  |
| Corrected Total | 60192.849 | 561 |  |  |  |
| a. R Squared = ,081 (Adjusted R Squared = ,076) | | | | | |

| **Dependent Variable: QCAE_AFFECTIVE EMPATHY** | | | | | |
| --- | --- | --- | --- | --- | --- |
| Source | Type III Sum of Squares | df | Mean Square | F | Sig. |
| Corrected Model | 1927.395^a^ | 3 | 642.465 | 26.462 | .000 |
| Intercept | 56064.530 | 1 | 56064.530 | 2309.221 | .000 |
| Sex | 323.763 | 1 | 323.763 | 13.335 | .000 |
| Age | 17.570 | 1 | 17.570 | .724 | .395 |
| Sex * Age | 9.752 | 1 | 9.752 | .402 | .526 |
| Error | 13547.431 | 558 | 24.279 |  |  |
| Total | 603834.000 | 562 |  |  |  |
| Corrected Total | 15474.826 | 561 |  |  |  |
| a. R Squared = ,125 (Adjusted R Squared = ,120) | | | | | |

| **Dependent Variable: QCEA_EMOTION CONTAGION** | | | | | |
| --- | --- | --- | --- | --- | --- |
| Source | Type III Sum of Squares | df | Mean Square | F | Sig. |
| Corrected Model | 183.658^a^ | 3 | 61.219 | 12.420 | .000 |
| Intercept | 7044.801 | 1 | 7044.801 | 1429.214 | .000 |
| Sex | 42.431 | 1 | 42.431 | 8.608 | .003 |
| Age | 1.171 | 1 | 1.171 | .238 | .626 |
| Sex * Age | 3.356 | 1 | 3.356 | .681 | .410 |
| Error | 2750.463 | 558 | 4.929 |  |  |
| Total | 80490.000 | 562 |  |  |  |
| Corrected Total | 2934.121 | 561 |  |  |  |
| a. R Squared = ,063 (Adjusted R Squared = ,058) | | | | | |

| **Dependent Variable: QCEA_PROXIMAL RESPONSIVITY** | | | | | |
| --- | --- | --- | --- | --- | --- |
| Source | Type III Sum of Squares | df | Mean Square | F | Sig. |
| Corrected Model | 211.220^a^ | 3 | 70.407 | 16.349 | .000 |
| Intercept | 7138.029 | 1 | 7138.029 | 1657.510 | .000 |
| Sex | 48.715 | 1 | 48.715 | 11.312 | .001 |
| Age | 9.330 | 1 | 9.330 | 2.166 | .142 |
| Sex * Age | 3.595 | 1 | 3.595 | .835 | .361 |
| Error | 2403.015 | 558 | 4.306 |  |  |
| Total | 84650.000 | 562 |  |  |  |
| Corrected Total | 2614.235 | 561 |  |  |  |
| a. R Squared = ,081 (Adjusted R Squared = ,076) | | | | | |

| **Dependent Variable: QCAE_PERIPHERAL RESPONSIVITY** | | | | | |
| --- | --- | --- | --- | --- | --- |
| Source | Type III Sum of Squares | df | Mean Square | F | Sig. |
| Corrected Model | 325.007^a^ | 3 | 108.336 | 22.958 | .000 |
| Intercept | 4672.998 | 1 | 4672.998 | 990.293 | .000 |
| Sex | 20.249 | 1 | 20.249 | 4.291 | .039 |
| Age | 69.358 | 1 | 69.358 | 14.698 | .000 |
| Sex * Age | .366 | 1 | .366 | .078 | .781 |
| Error | 2633.093 | 558 | 4.719 |  |  |
| Total | 43818.000 | 562 |  |  |  |
| Corrected Total | 2958.100 | 561 |  |  |  |
| a. R Squared = ,110 (Adjusted R Squared = ,105) | | | | | |

| **Dependent Variable: QCAE_COGNITIVE EMPATHY** | | | | | |
| --- | --- | --- | --- | --- | --- |
| Source | Type III Sum of Squares | df | Mean Square | F | Sig. |
| Corrected Model | 663.057^a^ | 3 | 221.019 | 3.882 | .009 |
| Intercept | 185947.665 | 1 | 185947.665 | 3266.067 | .000 |
| Sex | 141.857 | 1 | 141.857 | 2.492 | .115 |
| Age | 1.914 | 1 | 1.914 | .034 | .855 |
| Sex * Age | 10.612 | 1 | 10.612 | .186 | .666 |
| Error | 31768.731 | 558 | 56.933 |  |  |
| Total | 1953397.000 | 562 |  |  |  |
| Corrected Total | 32431.788 | 561 |  |  |  |
| a. R Squared = ,020 (Adjusted R Squared = ,015) | | | | | |

| **Dependent Variable: QCEA_PERSPECTIVE TAKING** | | | | | |
| --- | --- | --- | --- | --- | --- |
| Source | Type III Sum of Squares | df | Mean Square | F | Sig. |
| Corrected Model | 276.461^a^ | 3 | 92.154 | 3.856 | .009 |
| Intercept | 51826.362 | 1 | 51826.362 | 2168.306 | .000 |
| Sex | 7.985 | 1 | 7.985 | .334 | .563 |
| Age | 10.606 | 1 | 10.606 | .444 | .506 |
| Sex * Age | 8.595 | 1 | 8.595 | .360 | .549 |
| Error | 13337.192 | 558 | 23.902 |  |  |
| Total | 538857.000 | 562 |  |  |  |
| Corrected Total | 13613.653 | 561 |  |  |  |
| a. R Squared = ,020 (Adjusted R Squared = ,015) | | | | | |

| **Dependent Variable: QCEA_ONLINE SIMULATION** | | | | | |
| --- | --- | --- | --- | --- | --- |
| Source | Type III Sum of Squares | df | Mean Square | F | Sig. |
| Corrected Model | 124.484^a^ | 3 | 41.495 | 2.623 | .050 |
| Intercept | 41437.673 | 1 | 41437.673 | 2619.455 | .000 |
| Sex | 82.529 | 1 | 82.529 | 5.217 | .023 |
| Age | 3.509 | 1 | 3.509 | .222 | .638 |
| Sex * Age | 38.307 | 1 | 38.307 | 2.422 | .120 |
| Error | 8827.110 | 558 | 15.819 |  |  |
| Total | 446206.000 | 562 |  |  |  |
| Corrected Total | 8951.594 | 561 |  |  |  |
| a. R Squared = ,014 (Adjusted R Squared = ,009) | | | | | |
